# Measles Virus as an Oncolytic Immunotherapy

**DOI:** 10.3390/cancers13030544

**Published:** 2021-02-01

**Authors:** Christine E. Engeland, Guy Ungerechts

**Affiliations:** 1 Clinical Cooperation Unit Virotherapy, German Cancer Research Center (DKFZ), National Center for Tumor Diseases (NCT) and Department of Medical Oncology, University Hospital Heidelberg, 69120 Heidelberg, Germany; 2 Center for Biomedical Education and Research (ZBAF), Institute of Virology and Microbiology, Faculty of Health, School of Medicine, Witten/Herdecke University, 58453 Witten, Germany

**Keywords:** oncolytic virus, measles virus, cancer immunotherapy, vector engineering, vaccination, immune checkpoint blockade

## Abstract

**Simple Summary:**

Measles virus is currently under investigation as an innovative cancer treatment. The virus selectively replicates in and kills cancer cells. Furthermore, it can be genetically engineered to increase tumor specificity and therapeutic efficacy. Importantly, treatment with measles virus activates antitumor immune responses. A number of clinical trials using measles virus for cancer treatment have been completed or are ongoing. Future studies will further harness the possibilities of virus engineering and potential of combination immunotherapies to improve clinical outcome.

**Abstract:**

Measles virus (MeV) preferentially replicates in malignant cells, leading to tumor lysis and priming of antitumor immunity. Live attenuated MeV vaccine strains are therefore under investigation as cancer therapeutics. The versatile MeV reverse genetics systems allows for engineering of advanced targeted, armed, and shielded oncolytic viral vectors. Therapeutic efficacy can further be enhanced by combination treatments. An emerging focus in this regard is combination immunotherapy, especially with immune checkpoint blockade. Despite challenges arising from antiviral immunity, availability of preclinical models, and GMP production, early clinical trials have demonstrated safety of oncolytic MeV and yielded promising efficacy data. Future clinical trials with engineered viruses, rational combination regimens, and comprehensive translational research programs will realize the potential of oncolytic immunotherapy.

## 1. Introduction—Measles Virus for Cancer Therapy

**Figure 1:**
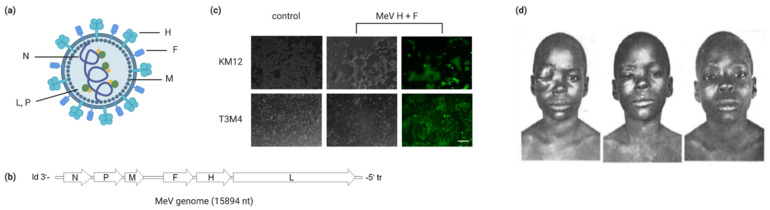
Measles as an oncolytic virus. (**a**) Schematic of the measles virus particle. The viral RNA genome is encapsulated by the nucleocapsid (N) protein and is associated with the viral polymerase (L, large protein) and its cofactor phosphoprotein (P), forming the ribonucleoprotein complex (RNP). The matrix (M) protein connects the RNP and the viral envelope. The surface glycoproteins hemagglutinin (H) and fusion (F) mediate receptor binding and cell fusion, respectively. (**b**) Schematic of the measles virus genome with open reading frames encoding the six structural proteins flanked by the 3′ leader (ld) and 5′ trailer (tr). (**c**) Syncytia formation as the typical cytopathic effect associated with measles virus infection. Human colorectal cancer (KM12, top) and pancreatic adenocarcinoma (T3M4, bottom) cells were transfected with plasmids encoding the MeV glycoproteins H and F as well as enhanced green fluorescent protein as reporter. Control cells were subjected to mock transfection. Phase contrast and fluorescence images were acquired with an Axiovert 200 microscope (Zeiss) at 36 h (KM12) and 12 h post-transfection (T3M4). Scale bar: 200 µm. (**d**) Lymphoma remission after measles infection. Left panel: The patient presented with orbital Burkitt’s lymphoma. Middle panel: The patient was infected with measles; the typical skin rash is visible. Right panel: Without specific anti-lymphoma treatment, the orbital mass resolved. Reproduced from Lancet 10 July 1971; 2 (7715): 105–106, with permission.

Measles virus (MeV) is a negative-strand RNA virus belonging to the family Paramyxoviridae, genus *Morbillivirus*. Its genome has a length of approximately 16 kb and encodes six structural and two non-structural proteins (Figure 1a,b). The viral glycoproteins hemagglutinin and fusion mediate receptor binding and fusion at the plasma membrane, respectively. While wild type MeV uses CD150/SLAM on lymphoid cells and epithelial nectin-4 as receptors, vaccine strains of MeV infect cells primarily via CD46 [1]. This is due to mutations in the receptor attachment protein hemagglutinin H in vaccine strain MeV, resulting in high affinity of H for CD46 [2,3,4,5,6]. MeV infection results in syncytia formation as typical cytopathic effect (Figure 1c).

Originally, the idea to treat cancer patients with MeV arose after case reports which linked measles infection to tumor remission [7]. One highly cited example relates to a boy suffering from Burkitt’s lymphoma [8] (Figure 1d). These experiments of nature inspired the idea of using MeV in cancer treatment. However, measles is a severe infectious disease [9]. Thus, employing a pathogenic strain of MeV in cancer therapy is out of question. Live attenuated MeV strains for vaccination were licensed in the 1960s and have a proven safety record [10]. Several years later, testing of Edmonston B measles vaccine strain derivatives for cancer treatment began. In many early studies, hematological malignancies were chosen as target entities [11,12,13,14]. This was supported by the natural lymphotropism of MeV. However, other malignancies including ovarian cancer [15] and glioblastoma [16] were soon found to also be sensitive to MeV oncolysis, while normal cells are spared [15,17].

Meanwhile, preclinical efficacy of oncolytic MeV has been demonstrated against a broad range of cancer entities (reviewed in [18]). In addition to Edmonston B derivatives, also the vaccine strains Moraten-Schwarz [19], Edmonston-Zagreb and AIK-C [20], rMV-Hu191 [21], as well as Leningrad-16 [22] have been shown to exert oncolytic effects in preclinical studies.

Thus, MeV is one of several oncolytic platforms currently developed for cancer therapy. Advantages of MeV include the excellent safety profile of the oncolytic vaccine strains and lack of genotoxicity, its immunogenicity, and especially the plethora of engineering possibilities offered by the MeV reverse genetics system. Specific challenges related to MeV include pre-existing antiviral immunity, the choice of preclinical models and manufacturing. These assets and drawbacks are discussed in more detail within this review article.

## 2. Measles Virus Oncotropism

Measles vaccine strain oncotropism correlates with CD46 overexpression on malignantly transformed cells [23]. Although viral entry occurs in benign cells and at low CD46 receptor density, a certain threshold of expression is required for syncytia formation and cell death [24]. In myeloma, CD46 upregulation has been associated with abnormal p53 [25]. The epithelial receptor for MeV, nectin-4 [26,27], is also a tumor marker which may render carcinomas of pancreatic [28], colorectal [29], and mammary [30] origin susceptible to MeV oncolysis. Post-transcriptional regulation of nectin-4 levels by miR-31 and miR-128 has been demonstrated in breast cancer and glioblastoma [31]. In certain EBV-associated B cell lymphomas, viral latency may promote upregulation of the MeV receptor CD150/SLAM [32].

On the post-entry level, the cellular interferon (IFN) response has been identified as a key determinant of sensitivity to oncolytic MeV across several tumor entities, including the NCI60 panel of cancer cell lines [33]. In adult T cell leukemia/lymphoma, resistance to MeV oncolysis was associated with IFN-β production, while sensitive cells did not produce IFN [34]. In mesothelioma and melanoma, effects of treatment with oncolytic MeV were found not to correlate with CD46 expression, but rather with defects in the IFN response [35,36]. Consistently, expression of retinoic acid inducible gene I (RIG-I) and IFN-induced protein with tetratricopeptide repeats 1 (IFIT1) [37] and IFN-induced transmembrane protein 1 (IFITM1) [38] have been suggested as correlates of relative resistance to MeV oncolysis. Kurokawa et al. have devised a gene expression signature designating constitutive IFN pathway activation to predict outcome of oncolytic MeV treatment [39]. Further, RSAD2/viperin, encoded by an IFN-stimulated gene (ISG), has been shown to inhibit release of MeV progeny in ovarian cancer models [40].

**Figure 2:**
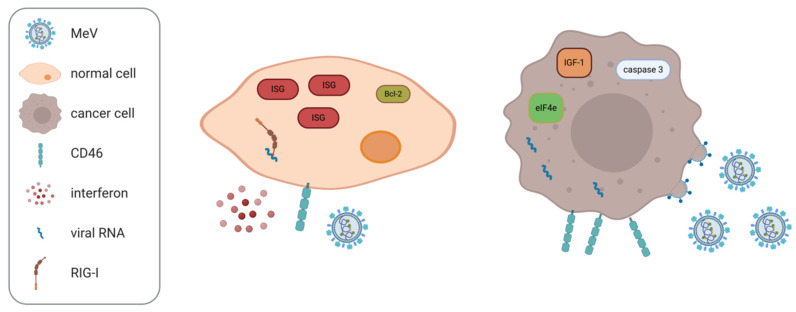
Measles virus oncotropism. Oncolytic measles virus (MeV) does not replicate productively in normal cells (**left**) in contrast to cancer cells (**right**). This oncotropism has been associated with differential expression of i.a. the depicted host cells factors. ISG: interferon-stimulated gene; RIG-I: retinoic acid-inducible gene I; IGF-1: insulin-like growth factor-I.

Aside from the cellular antiviral response, several additional cellular factors have been associated with sensitivity to MeV oncolysis. For instance, apoptosis regulators appear to play a role. Caspase 3 has been implicated in MeV-induced cancer cell death [41,42] and overexpression of Bcl-2 reduces MeV-induced cell death in B cell lymphomas [43]. More broadly, basic cellular processes such as protein translation are necessary for efficient MeV replication and thus tumor cell killing. Stimulating cellular translation by insulin-like growth factor-I (IGF-I) or forced expression of eIF4E increases efficacy of oncolytic MeV, while inhibitors of cap-dependent translation reduce MeV oncolysis [44]. Furthermore, it has been reported that integrity of lipid rafts is a prerequisite for oncolysis with the MV-Hu191 strain [21]. Determinants of MeV oncotropism are summarized in Figure 2.

Overall, oncolytic MeV acts via mechanisms distinct from other established cancer treatments. Accordingly, gemcitabine-resistant pancreatic adenocarcinoma cells are susceptible to MeV oncolysis [45] and chemotherapy-induced senescence does not abrogate oncolysis [46].

## 3. Combination Therapies

Nevertheless, monotherapy with oncolytic MeV will often be insufficient to cure advanced stage malignancies. Modern medical oncology builds on effective combination therapies. Therefore, measles virotherapy has been combined with other established cancer therapies such as radiation and chemotherapy (recently reviewed in [47]). Synergistic effects of oncolytic MeV and radiotherapy against glioblastoma were observed in vitro and in a xenograft model [48]. In vitro studies have also demonstrated successful combination of oncolytic MeV with chemotherapies such as paclitaxel [49], camptothecin [50], and gemcitabine [51]. Combination with the anti-epidermal growth factor receptor (EGFR) monoclonal antibody nimotuzumab was reported to result in increased antitumor efficacy in laryngeal cancer models [52].

Several small molecules have also been shown to enhance MeV oncolysis by modulating host cell factors. MeV infection is associated with heat shock protein (Hsp) 70 upregulation. Combination treatment with a Hsp90 inhibitor, resulting in increased Hsp70 expression [53,54], led to increased apoptosis [55]. Counteracting the IFN response, e.g., with janus-associated kinase (JAK) inhibitors such as ruxolitinib, enhances MeV replication in vitro [56]. Epigenetic modulation by histone deacetylase (HDAC) inhibition was also reported to increase efficacy of oncolytic MeV by preventing induction of ISGs in hepatocellular carcinoma [57], but by a different, so far unresolved mechanism in pancreatic adenocarcinoma [58]. As MeV spread and syncytia formation involves remodeling of the actin cytoskeleton, inhibition of Rho-associated coiled-coil forming kinase (ROCK) was tested during treatment of prostate, breast, and glioblastoma cancer cells with MeV, yielding increased viral replication, spread, and tumor cell killing [59]. Compounds which modulate cellular metabolism have also been tested in combination approaches. Blocking aerobic glycolysis with dichloroacetate was shown to increase cell death upon MeV treatment [60]. Furthermore, inducing autophagy has been suggested as a combination strategy to promote MeV oncolysis [61].

Even combination with other oncolytic viruses is conceivable. Along these lines, the combination of MeV with mumps virus showed increased efficacy in a human prostate cancer xenograft model [62].

## 4. Engineering Oncolytic MeV

**Figure 3:**
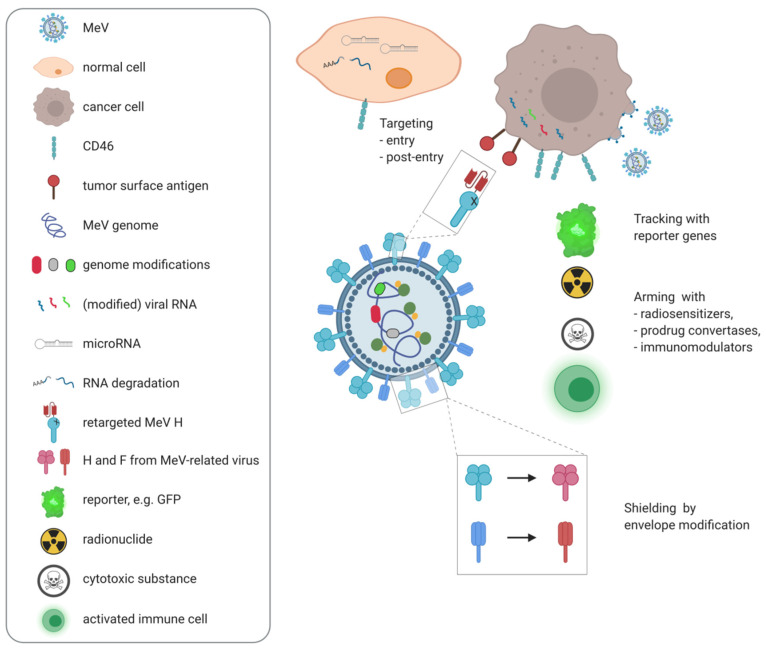
Engineering of oncolytic measles virus. Top: Targeting for increased tumor specificity can be achieved on the entry or post-entry level. For entry targeting, the viral attachment protein H can be mutated to ablate natural tropism and redirected by fusing targeting moieties such as antibody single-chain variable fragments to H (red). Post-entry targeting is achieved via target sites for microRNAs (gray) introduced into viral genes which are differentially expressed in malignant compared to healthy tissues, leading to degradation of the respective viral RNAs in normal cells. Middle: Viruses equipped with reporter genes encoding, e.g., fluorescent proteins (light green) can be used to track viral spread. To increase therapeutic efficacy, viruses can be armed with additional genes encoding radiosensitizers, prodrug convertases, or immunomodulators. Bottom: Shielding against neutralizing antibodies can be achieved by exchanging the viral envelope proteins for the counterparts from a related virus.

Purposeful modification of oncolytic MeV vectors to enhance virotherapy was enabled by development of a reverse genetics system for rescue of MeV from cloned cDNA [63]. This system allows for insertion of transgenes via additional transcription units equipped with MeV polymerase regulatory sequences [64]. These genes are then expressed in infected cells, i.e., within the tumor. A plethora of genetic engineering approaches has been pursued which are summarized in the following, and in Figure 3 (for recent reviews, see [47,65]).

### 4.1. Tracking Viral Replication and Spread

Initially, reporter genes were inserted for tracking of MeV replication. Carcinoembryonic antigen (CEA) and β-human chorionic gonadotropin (HCG) were selected, which can be measured in routine clinical laboratory testing [12]. Encoding the sodium iodide symporter, NIS, yielding MV-NIS, allowed for γ-camera imaging of iodine-123 (^123^I) or 99m-technetium uptake and also radiotherapy with ^131^I [66]. In later studies, MV-NIS was used for advanced imaging techniques, such as pinhole micro-single photon emission computed tomography/computed tomography (SPECT/CT) [67] and contrast-enhanced CT [68]. Recently, a recombinant MeV variant encoding a fluorescent reporter gene was used for intravital imaging of viral spread at single-cell resolution by two-photon microscopy [69].

Data from preclinical studies with MV-NIS have also been used to develop mathematical models of oncolytic virotherapy and its combinations. This has been devised as a means to rationalize testing of distinct dosing and scheduling regimens [70,71,72,73].

Valuable information was gained by employing viruses with reporter genes in clinical trials. After intraperitoneal administration of MV-CEA, dose-dependent increases in CEA levels were measured in peritoneal fluid and serum [74]. After intraperitoneal administration of MV-NIS, ^123^I SPECT/CT scans were positive in three of 13 ovarian cancer patients, confirming viral gene expression at the tumor site. Scans were positive in eight of 31 multiple myeloma patients receiving MV-NIS i.v. [75]. In both studies, positive scans were associated with higher virus doses.

### 4.2. Retargeting MeV

Virus engineering has not only enabled tracking viral spread, but also modifying its tropism to increase tumor specificity. Retargeting of MeV was accomplished by mutating the intrinsic receptor binding sites and fusing antibody single-chain variable fragments (scFv) to the C-terminus of the viral hemagglutinin [76]. Using this strategy, oncolytic MeV were targeted to the myeloma surface antigen CD38 [13], to CD20 for targeting of B cell malignancies [77], folate receptor (FR)-α for treatment of ovarian cancer [78], and EGFRvIII expressed in glioblastoma [79], among others (reviewed in [18]). A range of different targeting moieties beyond scFv has been employed, such as the cytokine interleukin (IL)-13 [80] or the urokinase plasminogen activator [81] for direction of viral tropism to their respective receptors. Successful targeting has also been achieved using integrin-binding peptides [82], DARPins [83] and cystine knot proteins [84]. Viral tropism can be redirected to specific cell populations within the tumor, including tumor-initiating cells [85], the tumor stroma [86], and vasculature [81].

A sophisticated means of viral entry targeting employs proteases expressed within the tumor microenvironment. The MeV fusion protein encompasses a furin cleavage site and requires proteolytic processing for activity. Replacing the furin cleavage site with sequences recognized by matrix metalloproteinases or the urokinase-type plasminogen activator can increase tumor specificity [87,88].

Tumor targeting on the post-entry level was achieved using microRNA target sites inserted into the untranslated regions (UTRs) of viral genes [89]. This concept exploits downregulation of specific microRNAs in malignant vs. benign cells, leading to virus restriction in healthy tissue while spread within tumor tissue is unimpaired.

Proof-of-concept was also obtained for using riboswitches to control oncolytic MeV. Insertion of a ligand-activated ribozyme into the UTR of the MeV fusion gene enabled regulation of MeV infectivity and spread by addition of the cognate small molecule [90]. Recently, a photocontrollable MeV variant was reported which harbors a split L protein for control of viral replication by blue light illumination [91].

### 4.3. Arming with Additional Therapeutic Genes

While these means of targeting aim at enhancing specificity of virotherapy, a number of genetic engineering approaches have been developed to increase antitumor efficacy, often referred to as “arming”. First arming strategies aimed at inducing bystander effects in combination radiotherapy and chemotherapy approaches. As mentioned above, MV-NIS allows for concentration of radioactive iodine in infected tumor cells [66].

MeV vectors encoding prodrug convertases were designed for local conversion of prodrugs into active chemotherapeutics. MeV encoding the purine nucleoside phosphorylase, which converts fludarabine into 2-fluoroadenine and 6-methylpurine-2’-deoxyriboside (MeP-dR) to 6-methylpurine, respectively, combined with prodrug administration improved outcome in lymphoma xenograft and immunocompetent murine colorectal cancer models [92,93]. Analogously, MeV was engineered to encode super cytosine deaminase (SCD), a fusion protein of yeast cytosine deaminase and yeast uracil phosphoribosyltransferase, which converts the prodrug 5-fluorocytosine (5-FC) to 5-fluorouracil (5-FU) [94,95,96,97].

Other engineering approaches to increase anti-tumor efficacy include insertion of a transgene encoding the proapoptotic protein BNiP3 [49] and the angiogenesis inhibitors endostatin and angiostatin to remodel the tumor microenvironment [98].

## 5. Immunovirotherapy

While early efforts in engineering oncolytic MeV mainly focused on maximizing direct tumor cell killing, there has been a recent shift from mainly oncolytic to mainly immunotherapeutic treatment strategies, spurred by the developments in cancer immunotherapy which have revolutionized medical oncology.

**Figure 4:**
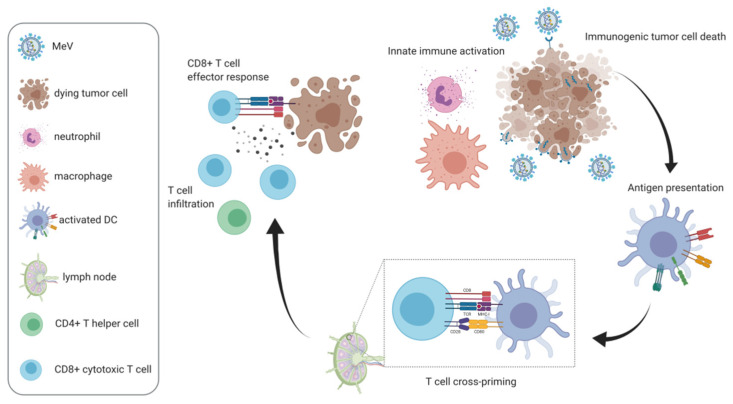
Measles virus as an oncolytic immunotherapy. Measles virus-mediated oncolysis has been shown to support different phases of the antitumor immune response: Oncolysis induces immunogenic cell death, which promotes dendritic cell activation, antigen presentation, and cross-priming of T cells. Measles virotherapy remodels the tumor microenvironment, thereby enhancing innate (macrophage repolarization and neutrophil degranulation) as well as adaptive antitumor immunity (T cell infiltration and CD8+ effector responses).

MeV oncolysis per se has pleiotropic effects on the anti-tumor immune response and supports all phases of the “cancer immunity cycle” (Figure 4; reviewed in [99]). MeV-induced cell death is immunogenic [100], induces a distinctive immunopeptidome [101], and promotes cross-priming of antitumor T cell responses by conventional and plasmacytoid dendritic cells [19,102]. MeV oncolysis has also been reported to increase tumor necrosis factor-related apoptosis-inducing ligand (TRAIL)-mediated cytotoxicity by myeloid and plasmacytoid DCs [103] as well as modulation of macrophages towards an antitumor phenotype [104]. Neutrophil activation also occurs, leading to secretion of IL-8, tumor necrosis factor (TNF)-α, monocyte chemoattractant protein (MCP)-1, and IFN-α, TRAIL expression, and degranulation [105], which may be beneficial or not depending on the tumor model [106].

These immunotherapeutic effects can be enhanced by insertion of immunomodulatory transgenes into the MeV genome (Table 1). Further, MeV can serve as a vector to deliver immunomodulators to the tumor site which can be highly toxic when administered systemically. The first immunomodulatory transgene reported in the context of many oncolytic viruses and also MeV was the granulocyte macrophage colony stimulating factor, GM-CSF [14]. In a lymphoma xenograft model, MV GM-CSF led to increased neutrophil infiltration, which correlated with tumor regression. Further immunomodulators have been shown to increase innate immune activation in the context of MeV oncolysis. A MeV vector encoding IFN-β was reported to induce immune infiltration and remodeling of the tumor microenvironment in mesothelioma xenografts [107]. MeV encoding the immunomodulatory neutrophil-activating protein (NAP) of *H. pylori* prolonged survival and induced a beneficial cytokine response in breast cancer xenograft pleural effusion and lung colonization models [108].

Introduction of the first fully immunocompetent mouse model of MeV oncolysis, MC38cea [93], was the prerequisite to further study immunomodulatory MeV vectors and demonstrate induction of tumor-specific adaptive immune responses. This model consists of murine colorectal adenocarcinoma MC38, syngeneic to C57BL/6 mice and stably expressing the carcinoembryonic antigen (CEA), which are susceptible to CEA-targeted MeV [93]. In this model, treatment with MV GM-CSF led to prolonged survival compared to control MV. Forty percent of treated mice experienced complete tumor remission and were subsequently protected from tumor re-engraftment, indicating a tumor vaccination effect. Further, treatment with MV GM-CSF enhanced intratumoral T cell infiltration as well as tumor-specific T cell responses [109].

To develop a second immunocompetent model of MeV oncolysis in C57BL/6 mice, B16 melanoma cells were transduced for stable expression of the CD20 surface antigen for treatment with CD20-targeted MeV. In this model, MeV vectors encoding immune checkpoint antibodies against cytotoxic T lymphocyte-associated-4 (CTLA-4) and programmed cell death-ligand 1 (PD-L1) prolonged survival compared to MeV encoding the antibody constant region only [110]. Combination with systemically administered antibodies against CTLA-4, PD-1, and PD-L1 has also demonstrated the therapeutic value of this approach [110,116]. In the MC38cea model, systematic comparison of transgenes targeted at different phases of the cancer immunity cycle—GM-CSF, IFN-γ induced protein 10 (IP-10), membrane-bound CD80, anti-CTLA-4, IL-12, and anti-PD-L1 identified the latter two as the most potent [111]. MeV encoding IL-12 induced complete tumor remissions in 90% of treated mice, which were mediated by CD8+ effector T cell responses. Oncolytic MeV vectors encoding an IL-15 superagonist mediated T and NK cell activation, but were less effective than MeV encoding IL-12 [112]. Bispecific T cell engagers (BiTEs) simultaneously bind CD3 on T cells and a tumor surface antigen, thereby redirecting T cells to tumor cells to mediate antitumor T cell cytotoxicity. Oncolytic measles viruses encoding BiTEs were shown to promote T cell infiltration and activation in syngeneic and patient-derived tumor models [113].

For induction of T cell responses against specific antigens, MeV can also be employed as a heterologous, highly immunogenic vaccine vector (reviewed in [117]). This strategy has been used to develop vaccines against a range of pathogens, including emerging SARS CoV-2 [118]. This strategy has been adopted in oncolytic immunotherapy by encoding tumor-associated antigens in the MeV vector. MeV vectors encoding ovalbumin (OVA) as model antigen or the tumor antigen claudin-6 either in native form or in association with lentivirus-like particles were shown to induce antigen-specific humoral and cellular immune responses in IFN-α receptor (IFNAR)-deficient, CD46-transgenic mice and prolong survival in B16-derived tumor models [114]. Employing OVA and the melanoma antigen tyrosinase-related protein-2 (TRP-2), MeV vectors encoding the full-length antigens or their respective immunodominant CD8+ epitope or epitope variants were generated. The epitope variants are either secreted or targeted to the proteasome. Using these MeV vectors, activation and dendritic cell-mediated priming of cognate T cells was demonstrated ex vivo [115].

As another modality of immunovirotherapy, combination of oncolytic MeV with adoptive transfer of antitumor immune effector cells such as NK cells [119] or CD8+ NKG2D+ cells [120] has been reported.

Importantly, antitumor immune activation by MeV oncolysis has not only been demonstrated in preclinical models. Clinical data also suggest augmentation of antitumor immunity by oncolytic measles virotherapy. In cutaneous T cell lymphoma, a shift towards a Th1-biased T cell population in lymphoma lesions was noted after treatment [121]. In four ovarian cancer patients treated with MV-NIS, IFN-γ and IL-4 responses against the tumor antigens FRα and IGF binding protein 2 (IGFBP2) were detected by ELISPOT [122]. Increases in IFN-γ ELISPOT counts against cancer testis antigens were also observed in the majority of tested multiple myeloma patients treated with MV-NIS [123]. The myeloma patient with an exceptional response to MV-NIS had a high mutational load and high baseline T cell responses against several tumor antigens, which remained stable after virotherapy.

Of note, the clinical trials published thus far tested oncolytic MeV not encoding any additional immunotherapeutic payloads. Perhaps the fraction of patients showing immunological responses and overall therapeutic efficacy can be increased with novel immunomodulatory oncolytic MeV.

## 6. Antitumor vs. Antiviral Immunity

However, immune stimulation in the context of oncolytic virotherapy may hamper overall efficacy by premature viral clearance [124]. Though conferring a safety advantage, the antiviral immune response and specifically high measles seropositivity in the general population is one of the main reservations against using MeV for oncolytic virotherapy. Therefore, multiple strategies have been devised to circumvent anti-viral immunity. Substitution of the P/V/C and also N and L genes of attenuated oncolytic strains for their wild type counterparts has been shown to dampen the cellular IFN response and increase viral spread [125,126]. These variants resulted in higher progeny titers, increased viral gene expression, and cell killing in presence of interferon or in interferon-competent cells. Mutation of common antibody epitopes in the MeV envelope glycoproteins allows for evasion of virus neutralization in serum [127]. By exchanging the glycoproteins for their counterparts from a related morbillivirus, canine distemper virus, an envelope chimeric MeV was generated which showed similar replication kinetics and oncolytic properties as unmodified MeV, but was not neutralized by human MeV-immune sera [128]. However, these approaches may compromise safety. As alternatives, different “shielding” approaches have been developed to protect oncolytic MeV from antibody-mediated clearance.

One approach is to employ cell carriers to “deliver” oncolytic MeV to the tumor site. Successful tumor delivery by heterofusion of infected carrier cells and tumor cells was first demonstrated for infected monocytes, endothelial cells, and stimulated human peripheral blood cells. This allowed for effective oncolysis after i.v. or i.p. administration after passive immunization in xenograft models [129]. A range of different cell types have been employed as carriers, including T cells [130], cytokine-induced killer cells [131], mesenchymal stem cells [132], mesenchymal stromal cells [133], and also irradiated myeloma cells [134].

As an acellular shield, the scavenger receptor ligand polyinosinic acid can be used to prevent MeV sequestration by hepatic Kupffer cells after i.v. administration [135]. This was shown to enhance oncolytic efficacy in a nude mouse model. Multi-layer coating with ionic polymers and graphene oxide sheets [136] have also been reported as a means to protect MeV from premature clearance [137]. These modifications did not compromise infection of tumor cells and even enhanced oncolytic effects. Administration of UV-inactivated MeV as a decoy virus has been suggested as a means to sequester antiviral antibodies prior to treatment [138].

Instead of modifying the oncolytic agent, immune modulation in the patient has been envisaged to enable measles virotherapy. Clinically approved multidose cyclophosphamide regimens were shown to dampen both primary and secondary antibody responses to MeV [139]. Although pre-existing immunosuppression in advanced stage cancer and especially low antibody levels in myeloma patients were anticipated, cyclophosphamide was also tested in one cohort of the Phase I trial of MV-NIS for advanced multiple myeloma [75]. Clinical data in this regard are still limited, but so far no clear correlation between anti-measles immunity and therapeutic efficacy has been noted.

## 7. Preclinical Models

The conundrum of balancing antiviral immunity and antitumor immunity exemplifies the challenge to identify appropriate models for preclinical development of oncolytic MeV. Measles is a primate-adapted virus, thus rodents and other small animals commonly used in research are non-susceptible to the virus. CD46-transgenic, IFNAR-knockout (IFNAR^−/−^ CD46Ge) mice which are supposed to mimic MeV replication and spread in humans are commonly used for study of MeV vaccines [117] and have also been used for testing of oncolytic MeV vaccines [114]. However, it remains unclear how the IFNAR^−/−^ phenotype affects outcome of virotherapy. Syngeneic transplantable tumor models in fully immunocompetent mice have been widely adopted [99]. While these models have enabled proof-of-concept studies, they fail to recapitulate the genetic makeup, heterogeneity and evolution of human cancers. To address these issues, human precision cut liver slices [20], clinical samples [140], and patient-derived xenografts [113] have been used in preclinical testing of measles virotherapy. Successful targeting of cancer-initiating cells in patient-derived cultures, including glioma stem cells from neurospheres [141] and colorectal cancer tumor spheroids [85] have been reported. To address specific tumor niches, orthotopic models such as breast cancer pleural effusion [142] and intracranial glioblastoma models [116,141] have been studied, demonstrating efficacy of oncolytic MeV also in advanced preclinical models.

## 8. Pharmacokinetics and –Dynamics

In preparation of clinical trials, several preclinical toxicology and pharmacokinetic studies have been carried out in mice and non-human primates [143,144,145,146,147]. These studies confirmed safety of intravenous injection of up to 10^8^ and 4 × 10^8^ TCID_50_/kg oncolytic MeV in IFNAR^−/−^ CD46Ge and squirrel monkeys, respectively. Further, intraventricular injection of oncolytic MeV into the cerebrospinal fluid of IFNAR^−/−^ CD46Ge mice [147] and intrahepatic injection of prodrug convertase-armed oncolytic MeV in IFNAR^−/−^ CD46Ge mice and rhesus macaques [146] were tolerated. Depending on the model, different pharmacokinetics and dose–response relationships were observed. Notably, despite detection of viral RNA, no significant shedding of infectious virus was reported.

This holds true in clinical settings. Saliva and urine samples were free of infectious virus after i.p. administration of MV-CEA in ovarian cancer patients [74]. Up to 10^9^ TCID_50_ i.p. and 10^11^ TCID_50_ i.v. have been administered with manageable side effects [75,122]. The available clinical data also suggest a dose–response relationship, with higher doses associated with more favorable outcome.

## 9. Early Clinical Trials with MeV

Clinical trials in oncology typically enroll patients after failure of established therapies. In the first clinical trial with oncolytic MeV, patients with therapy-resistant or relapsed cutaneous T cell lymphomas received intralesional injections of Edmonston-Zagreb measles vaccine. As a safety measure, IFN-α was administered prior to treatment. Treatment was well tolerated and tumor regressions, also of non-injected lesions, were observed. Serial biopsies showed intralesional viral replication and favorable changes in the intralesional T cell populations [121].

Quite a high number of subsequent trials were conducted at Mayo Clinic in patients with very different cancer entities including ovarian cancer [74,122] (NCT02068794; NCT00390299), glioblastoma multiforme (NCT00390299), medulloblastoma (NCT02962167), mesothelioma (NCT01503177), breast cancer (NCT04521764), head and neck squamous cell carcinoma (NCT01846091), malignant peripheral nerve sheath tumors (NCT02700230), bladder cancer (NCT03171493), and multiple myeloma (NCT00450814; NCT02192775) using Edmonston B-derived attenuated MeV. These Phase I/II trials showed that MeV administration through all investigated routes including intraperitoneal, intracranial, intratumoral, intrapleural, and intravenous administration is safe, feasible, and may lead to a favorable outcome compared to expected median survival in the treated patient population [74,122]. In patients with multiple myeloma, treatment with oncolytic MeV led to transient drops in serum free light chains as myeloma marker in several patients. One patient experienced a durable complete remission which is still ongoing to date [75,148].

## 10. Translational Considerations, Perspectives, and Conclusions

As a consequence of the observed dose–response relationships, highest feasible doses are administered in current trials. However, large-scale manufacturing of the required high-titer, highly purified good manufacturing practice (GMP)-grade recombinant MeV remains challenging [149], despite development of processes including production in serum-free cell culture, tangential flow filtration, and diafiltration [150,151,152]. Nevertheless, these efforts seem worthwhile, given the versatility of MeV as an oncolytic vector platform [47], the excellent safety record of MeV vaccines [10], as well as the biosafety profile [153] and genetic stability [154] of recombinant MeV.

As outlined above, several rational combination approaches to cancer immunovirotherapy employing MeV and different immunomodulators will be under clinical investigation in the future. Other OVs have already been combined successfully with immune checkpoint inhibitors [155] in clinical trials. Moreover, clinical translation of second-generation MeV engineered to encode relevant immunomodulators as illustrated above will most likely further improve clinical outcomes.

Moving forward in this direction, it will be decisive to validate predictive markers of response and resistance in a clinical setting. These markers should not only incorporate tumor cell characteristics, but also signatures of antitumor immune activation. By defining criteria of successful immunovirotherapy, these results will also assist in prioritizing the most effective therapeutic payloads and combination therapies. Towards this end, even early stage clinical trials must encompass comprehensive correlative research programs to accelerate the advancement of effective immunovirotherapies.

## Figures and Tables

**Table 1:** Immunomodulatory oncolytic MeV. Overview of immunomodulatory transgenes that have been encoded in MeV, their anticipated immunological effects in the context of MeV oncolytic immunotherapy, and the outcome of the respective preclinical studies. GM-CSF: granulocyte–macrophage colony-stimulating factor; IFN: interferon; NAP: neutrophil activating protein; CTLA-4: cytotoxic T lymphocyte antigen-4; PD-L1: programmed cell death 1-ligand 1; Th: T helper cell; T_eff_: effector T cell; T_reg_: regulatory T cell; IL: interleukin; AICD: activation-induced cell death; BiTE: bispecific T cell engager; TAA: tumor-associated antigen; IFNAR: IFN-α receptor.

Immunomodulator	Anticipated Immunological Effects	Preclinical Data
GM-CSF	Dendritic cell activation and maturation; activation of monocytes, macrophages, neutrophils, NK cells	SCID model: increased antitumor efficacy, increased neutrophil infiltration [14] Immunocompetent model: increased antitumor efficacy, increased T cell infiltration, stronger tumor-specific T cell responses, rejection of tumor re-engraftment [109]
IFN-β	Enhanced antitumor response via innate and adaptive effector mechanisms	Athymic nude mouse model: increased CD68+ macrophage infiltration, reduced microvessel density; delayed tumor progression, prolonged survival [107]
*H. pylori* NAP	Inflammatory response, promotion of Th1-polarized immune responses	Athymic nude mouse model: prolonged survival, neutrophil infiltration, secretion of Th1-promoting cytokines [108]
Anti-CTLA-4, anti-PD-L1	Enhanced antitumor T cell response	Immunocompetent mouse model: delayed tumor progression, prolonged survival, increased T_eff_/T_reg_ ratio, increased tumor-specific IFN-γ response [110]
IL-12	Activation and recruitment of T cells and NK cells	Immunocompetent mouse model: increased survival rates (CD8+-dependent), rejection of tumor re-engraftment, increased tumor-specific IFN-γ response, expression of effector cytokines, increased T cell infiltration, decrease in NK cells, increased proportion of activated CD8+ T cells and NK cells [111]
IL-15 superagonist	Activation of T cells and NK cells without induction of AICD	Immunocompetent mouse model: increased CD8+ T cell and NK cell infiltration and activation, antitumor efficacy inferior to MeV encoding IL-12 [112]
BiTEs	Recruitment of T cells, enhanced T cell antitumor cytotoxicity	Immunocompetent mouse model: increased T cell infiltration, prolonged survival, induction of tumor-specific immunity Patient-derived xenograft models: prolonged survival [113]
TAA	Priming and activation of TAA-specific T cells	IFNAR^-/-^ CD46 transgenic mouse model: Induction of humoral and cellular responses against TAA, reduced tumor nodules and prolonged survival in lung colonization experiment [114] Ex vivo assays: Priming and activation of TAA-specific T cells [115]

Italic: bacterial taxa.
